# Real-Time Automated Solubility Screening Method Using Deep Neural Networks with Handcrafted Features

**DOI:** 10.3390/s23125525

**Published:** 2023-06-12

**Authors:** Minwoo Jeon, Geunhyeok Yu, Hyundo Choi, Gahee Kim, Hyoseok Hwang

**Affiliations:** 1 Department of Software Convergence, Kyunghee University, Yongin 17104, Republic of Korea; alsdn2530@khu.ac.kr (M.J.); geunhyeok@khu.ac.kr (G.Y.); 2 Material Research Center, Samsung Advanced Institute of Technology, Samsung Electronics, Suwon 16678, Republic of Korea

**Keywords:** solubility measurement, automated solubility screening, handcrafted feature, deep neural networks, support vector machine

## Abstract

Solubility measurements are essential in various research and industrial fields. With the automation of processes, the importance of automatic and real-time solubility measurements has increased. Although end-to-end learning methods are commonly used for classification tasks, the use of handcrafted features is still important for specific tasks with the limited labeled images of solutions used in industrial settings. In this study, we propose a method that uses computer vision algorithms to extract nine handcrafted features from images and train a DNN-based classifier to automatically classify solutions based on their dissolution states. To validate the proposed method, a dataset was constructed using various solution images ranging from undissolved solutes in the form of fine particles to those completely covering the solution. Using the proposed method, the solubility status can be automatically screened in real time by using a display and camera on a tablet or mobile phone. Therefore, by combining an automatic solubility changing system with the proposed method, a fully automated process could be achieved without human intervention.

## 1. Introduction

Measuring solubility is essential for designing and optimizing several areas of chemical research and industry, ranging from pharmaceutical applications to formulations and materials science [1,2,3]. All chemical reactions begin with the reactants uniformly dissolved in a solvent, so the accurate measurement of solubility is important. Solubility data also provide important information on the appropriate solvent and amount of solvent for recrystallization in the work-up and purification steps [4]. Solubility can be measured through three methods: optical density measurements using spectrometers, turbidity measurements using nephelometers and turbidimeters [5], and high-performance liquid chromatography (HPLC) measurements [6]. These methods can measure solubility with high accuracy, but cannot measure solubility in real time or automatically.

With the increase in automated processes, there has been a recent emergence of the need for real-time automatic solubility measurements. For example, in the semiconductor manufacturing process, it is necessary to determine whether the solute in the organic solvent has dissolved [7,8]. Currently, this measurement process is visually verified by humans and is not fully automated. Performing solubility measurements automatically and in real time is essential for achieving end-to-end automated processes. Several benefits in implementing end-to-end automation exist. First, it provides real-time data that can be used to optimize the process, improve efficiency, and reduce costs [9]. It can also help reduce human error and improve accuracy. Furthermore, it can aid in identifying potential problems before they escalate into major issues [1]. To achieve end-to-end automation, a method comprising algorithms that can be generalized to various measurement environments and experimental settings is required. As solubility is defined as the amount of compound that dissolves in a given amount of solvent at a given temperature when the system is in equilibrium, the presence or absence of undissolved solutes in the solution can be determined based on computer vision algorithms.

Therefore, vision methods based on deep neural networks (DNNs) are used to automatically obtain solutions in real time. There are two approaches: one extracts handcrafted features, whereas the other performs end-to-end learning by inputting only images, which automatically determine solubility. End-to-end learning has become popular in various classification tasks because it can automatically extract features through a convolutional neural network (CNN) without directly designing a pipeline. However, it may not be the best option for specific tasks, such as solubility screening, which we address in this study. In this task, the dataset consists of real-world industrial solutions; therefore, the amount of labeled data is small. In addition, because it is necessary to detect even small undissolved particles to determine solubility, designing the pipeline and finely adjusting it to extract features are more efficient.

**Figure 1:**
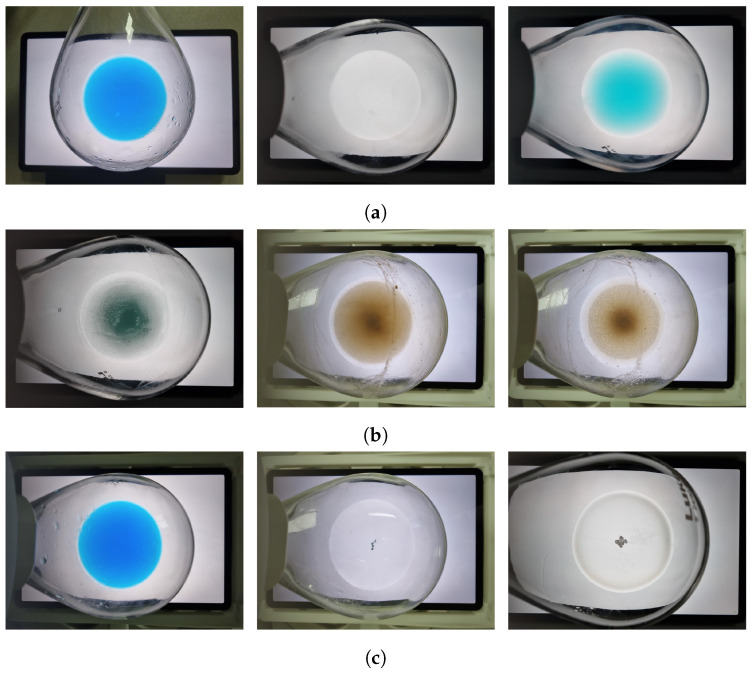
Examples of classified solution images. (**a**) Cases classified as DS. (**b**) Cases classified as US1. (**c**) Cases classified as US2.

We propose a method that utilizes computer vision to extract handcrafted features and measure solubility automatically in real time using only simple devices, such as an off-the-shelf camera and tablet. The solution in the flask was captured on a tablet displaying either a white background image or a checked background. We extracted eight features from the white background image and one from the checked background image. For the white background image, the captured solution image was analyzed by dividing it into a grid to analyze the pixel distribution in each grid and dividing it into a radial form to analyze the pixel distribution in each radial region, as well as to detect any undissolved particles. For the checked background image, the superposition between the check pattern of the actual background image and that of the captured solution image was analyzed. Using the extracted handcrafted features, we train the DNN model to classify a solution into three categories based on its solubility. The network that performs these tasks is called the ‘automated solubility screening (ASS) Net.’ The classification cases are shown in Figure 1. The dissolved state (DS) is when the solute is completely dissolved and clear, undissolved state 1 (US1) is when the solute is cloudy because it is barely dissolved, and undissolved state 2 (US2) is when the solute remains in the solution in the form of particles. The dataset consisted of flask images captured on a white background and checked background images, and the solutions are labeled into three categories based on their solubility. The dataset includes solutions that humans find difficult to differentiate because of the presence of small amounts of undissolved particles. The primary contributions of this study are as follows:

- We present a novel method that automatically determines the solubility of a solution by analyzing two images from a conventional camera and tablet, making the screen system easy to configure.
- The proposed DNN-based method with vision-based handcrafted features allows accurate screening of the solubility of a solution in real time.
- Our method can measure turbidity and classify various artifacts, such as undissolved fine particles and a large number of particles in the solution, even if the solution is very turbid or has Moiré.
- Our handcrafted features can also be applied to other classifiers, such as support vector machines (SVMs) [10].

The remainder of this paper is organized as follows. Section 2 introduces the existing solubility measurement methods and presents a study that uses computer vision and deep learning to measure solubility. Section 3 explains the methods used to extract the handcrafted features and classifiers. Section 4 describes the construction of the dataset and analysis of the experimental results.

## 2. Related Work

The accurate measurement of solubility is crucial in research and industry, and various methods have been developed to achieve this goal [2,3]. Two main methods are available for measuring solubility. One is based on measuring the amount of light scattered by the particles in a solution using nephelometers [11,12] and turbidimeters [13,14], the other involves using ‘excess solid’ or ‘excess solvent’ methods [1]. The former, which uses light transmission and scattering to determine whether a solution is soluble, requires separate sampling and preparation steps. Therefore, it is not possible to analyze the solution in real time. The excess solid method involves adding a solute to the solvent until it is saturated, filtering out any undissolved solids, and measuring its weight to determine its solubility. An example is HPLC [6,15,16,17]. 

HPLC is a commonly used and reliable method for the automated analysis of solutions; however, it requires reference materials for testing and calibration for automation. The excess solvent method involves adding a solvent to the solute until it is completely dissolved and measuring the amount of dissolved solute to determine solubility. One example is liquid–liquid extraction (LLE) [18,19]. LLE is a simple, selective, and versatile method for determining solubility. However, it is difficult to automate because it requires large amounts of solvent, careful pH control, and potential interference from co-extracted substances. Furthermore, the methods introduced thus far are invasive, such as collecting samples for solubility measurements or directly injecting the analyzer into the sample. However, invasive methods cannot measure solubility in real time.

Active research is being conducted to automatically measure solubility using computer vision and deep learning. Shiri et al. [20] presented an end-to-end automated solubility measurement method using a webcam, robot, and system that automatically doses liquids and solids. It does not require a human operator and can determine dissolution in real time using a non-invasive method. However, a limitation exists in that the region of interest (ROI) is manually selected by the user; therefore, it must be used by someone familiar with the method. In addition, this method measures turbidity using the average brightness of the preselected ROI and uses it to measure solubility. Therefore, it is highly affected by the brightness of the surrounding environment, and it is difficult to accurately judge whether it is dissolved. In Reference [21], Pizzuto et al. proposed an end-to-end cascaded neural network model that photographs the solution in the vial, obtains ROIs with Mask R-CNN [22], and determines whether the solute is dissolved in a specific solvent through a CNN [23]. This method does not require the user to manually select the ROI because it is selected by the mask R-CNN. In addition, using an end-to-end learning model, the input data can be directly mapped to the output prediction without an explicit feature engineering process. However, this approach cannot accurately analyze the factors that determine the classification, as it relies on learning its own internal representation of the input data. Moreover, because features are obtained through a CNN, it is difficult to precisely detect solutes that exist in particle form without dissolution.

## 3. Proposed Method

### 3.1. Method Overview

**Figure 2:**
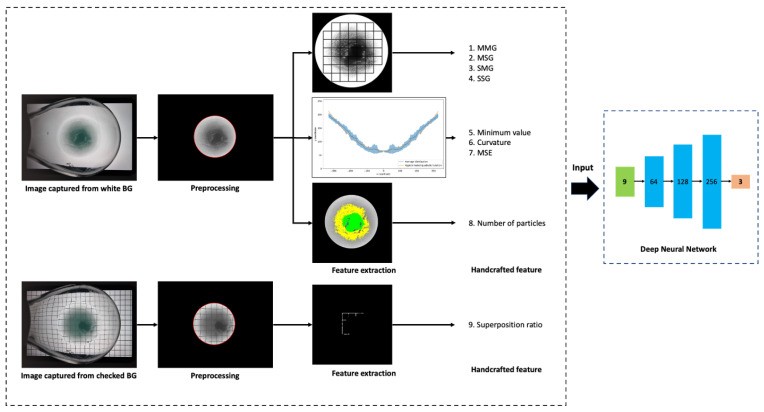
Overall process of the automated solubility screening method.

An overview of ASSNet, our automated solubility screening method, is shown in Figure 2. First, we captured an image of the flask with a tablet’s background image displayed. Since we needed to analyze the solution, we set the circular region corresponding to the solution as the ROI in the captured images. We then applied various computer vision algorithms to the ROI images to extract the handcrafted features. From the white background image, we analyzed the grid homogeneity, radial profile, and particle amount to extract eight features. From the checked background image, we analyzed the superposition of the checked background and captured images to extract a single feature. Grid homogeneity analysis (GHA) uses a white background image and can reveal both undissolved solutes and the cloudiness of the solution. Radial profile analysis (RPA) and particle amount analysis (PAA) use white background images to detect undissolved solutes. Superposition analysis (SA) uses a checked background image to determine the turbidity of a solution by quantifying the extent to which the check pattern is obscured. We used the nine extracted features to train a DNN and classified the images into three categories according to their solubility.

### 3.2. Preprocessing

The use of the entire captured image to analyze solubility is inefficient. Therefore, a preprocessing step was performed to set the ROI corresponding to the solution and perform masking. The Hough circle algorithm [24] was used to extract the ROIs because the solution in the flask was captured in a circular shape.

The circle closest to the image’s center is determined to be the final circle after several circles have been detected by lowering the threshold of the Hough circle algorithm. This method allows accurate solution detection, even for transparent solutions.

**Figure 3:**
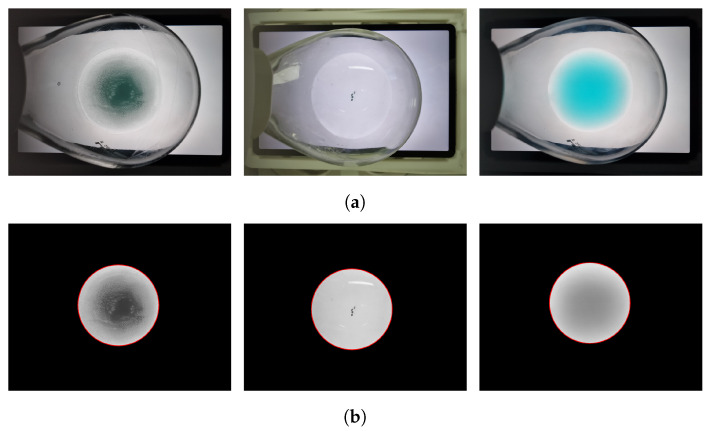
A series of preprocessing steps. (**a**) Images of the solution captured on the background image. (**b**) ROI masking images.

Finally, it was possible to determine the radius and center coordinates of the circle corresponding to the ROI in the image. Only the area corresponding to the ROI was left in the image after a masking operation, and the area that did not belong to the ROI had its pixel intensity set to zero. This enabled us to focus our analysis solely on the ROI, accelerating the calculations and enhancing the performance. The sequence of preprocessing steps is shown in Figure 3.

### 3.3. A Moiré Removal Process

We encountered a significant Moiré pattern issue in the captured images of the solution because of the method used to capture the solution on a tablet screen. Moiré patterns [25] commonly occur when capturing displays create a mismatch between the pixel pattern of the display and the sensor pattern of the camera. Moiré patterns are captured as black dots or lines in the image. As we used a method that detects even the finest particles in a solution, this is a critical problem.

**Figure 4:**
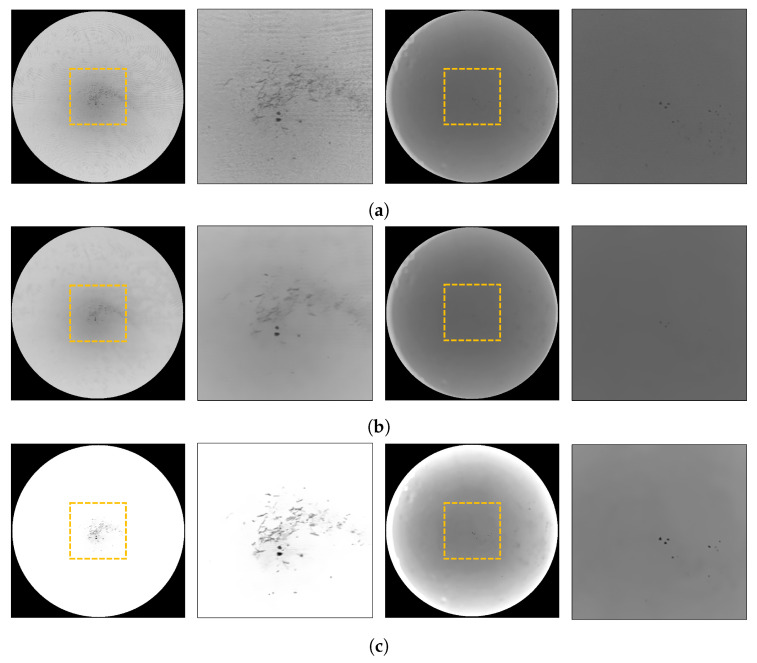
Moiré removal processes. The left panel is a solution containing undissolved fine particles captured with a Moiré pattern. The right panel is a colored solution containing undissolved fine particles. The yellow box represents the magnification region. (**a**) The original ROI image. (**b**) The result of applying the non-local means filter directly to the original ROI image. (**c**) The result of adjusting the scale and contrast of the original ROI image and applying the non-local means filter.

To solve the Moiré pattern problem, we applied a non-local means filter [26] to the ROI. However, when dealing with colored solutions, fine particles were removed along with the Moiré pattern, as shown on the right in Figure 4b. To address this problem, we increased the scale of the ROI image and enhanced the contrast before applying a non-local means filter. This is expressed as follows:

(1) $$dst^{\prime }=\mathrm{clip}\left(\left(\beta \times src\right),\;0,\;255\right),$$

(2) $$dst=\mathrm{clip}(\left(1+\alpha \right)\times dst^{\prime }-128\times \alpha ,\;0,\;255),$$

where α is 1.0 and β is 1.2. Therefore, we treated the colored solution as if it was a colorless liquid. As shown in Figure 4c, the Moiré pattern was effectively removed, leaving the fine particles intact.

### 3.4. Feature Extraction

We designed a pipeline to extract the handcrafted features. The pipeline performs GHA, RPA, and PAA on a solution image captured on a white background. Additionally, SA was performed on the solution image captured on the checked background. The features extracted through pipeline analysis are listed in Table 1.

#### 3.4.1. Grid Homogeneity Analysis

**Figure 5:**
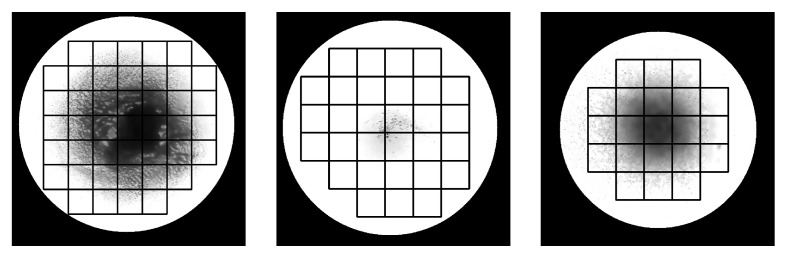
Examples that visualize the grid used to calculate the intensity distribution of pixels within the ROI area.

GHA is the process of extracting features through the pixel intensity distribution of the ROI identified as the solution. As described in Section 3.2, the solution is extracted as a circular ROI, and the center and radius of the circle can be calculated. Based on the center and radius of the circle, the coordinates of the square that circumscribes the circle can be determined; this square is then divided into a grid of uniform size. If the distance between the four corners of the grid and the center of the circle is less than the radius of the circle, the grid is considered to be within the circle. Using this method, the solution area can be divided into grids, as shown in Figure 5.

The mean of mean of grid (MMG), mean of standard deviation of grid (MSG), standard deviation of mean of grid (SMG), and standard deviation of standard deviation of grid (SSG) were obtained by analyzing the pixel distribution within each grid; these features were used in the analysis. The MMG, MSG, SMG, and SSG were calculated using the following equations:

(3) $$\mathrm{MMG}=\frac{1}{m}\sum_{i=1}^{m}\left(\frac{1}{n^{2}}\sum_{u=0}^{n-1}\sum_{v=0}^{n-1}G\left(u,v\right)\right),$$

(4) $$\mathrm{MSG}=\frac{1}{m}\sum_{i=1}^{m}\left(\sqrt{\frac{1}{n^{2}}\sum_{u=0}^{n-1}\sum_{v=0}^{n-1}{\left(G\left(u,v\right)-\bar{G(u,v)}\right)}^{2}}\right),$$

(5) $$\mathrm{SMG}=\sqrt{\frac{1}{m}\sum_{i=1}^{m}{\left(\frac{1}{n^{2}}\sum_{u=0}^{n-1}\sum_{v=0}^{n-1}G\left(u,v\right)-\bar{\frac{1}{n^{2}}\sum_{u=0}^{n-1}\sum_{v=0}^{n-1}G\left(u,v\right)}\right)}^{2}},$$

(6) $$\mathrm{SSG}=\sqrt{\frac{1}{m}\sum_{i=1}^{m}\left(\left(\sqrt{\frac{1}{n^{2}}\sum_{u=0}^{n-1}\sum_{v=0}^{n-1}{\left(G\left(u,v\right)-\bar{G(u,v)}\right)}^{2}}\right)-\bar{\left(\sqrt{\frac{1}{n^{2}}\sum_{u=0}^{n-1}\sum_{v=0}^{n-1}{\left(G\left(u,v\right)-\bar{G(u,v)}\right)}^{2}}\right)}\right)},$$

where *n* represents the pixel size of the width or height of the grid, with a value of 80, *m* is the total number of grids, and $\bar{.}$ denotes the mean. G(u,v) denotes the intensity value of a specific pixel within a grid.

#### 3.4.2. Radial Profile Analysis

**Figure 6:**
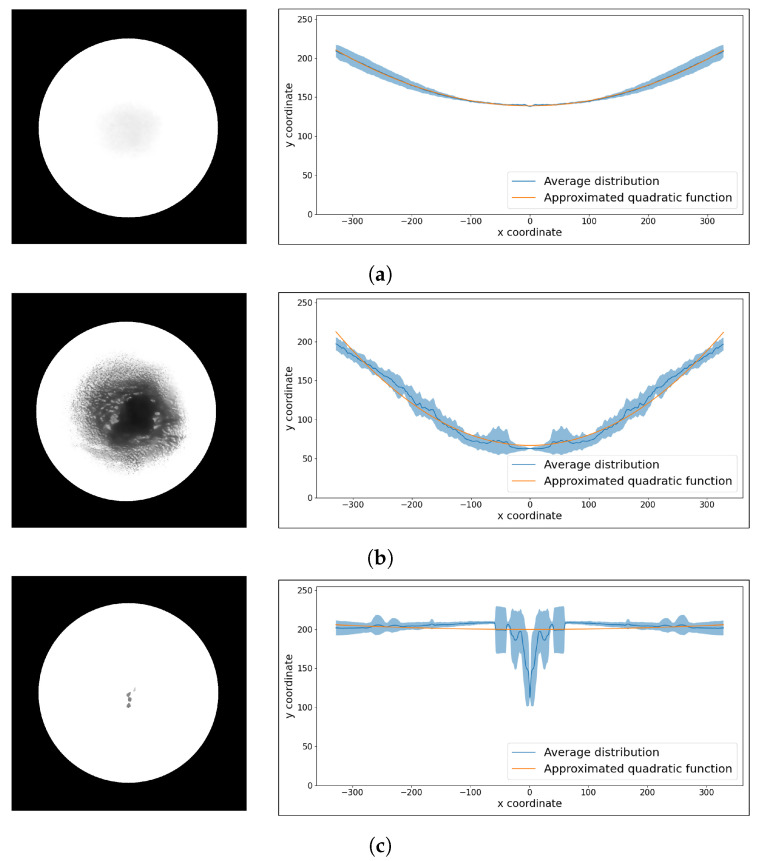
The left panels show the ROI images of solutions. The radial profile shows the average of the 12 distributions and a quadratic function approximating the average distribution. Additionally, the colored areas correspond to deviations from the 12 distributions. (**a**) DS, (**b**) US1, (**c**) US2 case example.

RPA is a method for extracting features by analyzing the distribution of pixel intensities corresponding to the diameter of an ROI while rotating every 30${}^{{}^{\circ}}$ around the center of the image. First, the center coordinates of the ROI circle and image were aligned using a transformation matrix. Subsequently, 12 radial distributions were obtained by rotating the image at 30${}^{{}^{\circ}}$ intervals around its center coordinates. The means of the 12 radial distributions were calculated and approximated using a quadratic function ($ax^{2}+c=0$). The curvature associated with ‘a’ of the quadratic function, the minimum value corresponding to ‘c’ of the quadratic function, and the mean squared error (MSE) between the quadratic function and the average value are used as features. The ROI images and their corresponding distributions for the DS, US1, and US2 cases are shown in Figure 6. The DS case, as shown in Figure 6a, has a gently sloping curvature for the approximated quadratic function. Small deviations between the 12 distributions and the average distribution indicate that the MSE value is small. US1, shown in Figure 6b, has a large curvature and a small minimum value for the approximated quadratic function. The US2 case, shown in Figure 6c, exhibits a large deviation among the 12 distributions owing to the presence of particles, resulting in a large difference between the average distribution and the approximated quadratic function. Consequently, the MSE value was large.

#### 3.4.3. Particle Amount Analysis

**Figure 7:**
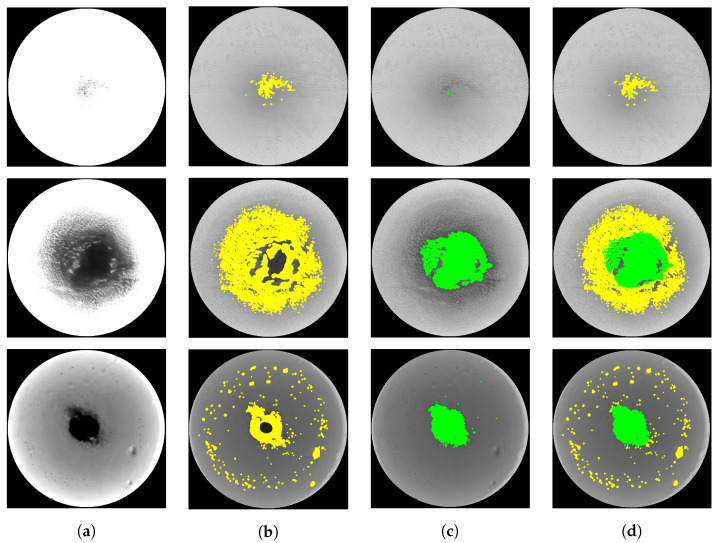
A series of PAA processes were performed on a solution with varying amounts of undissolved solute. (**a**) The ROI image. (**b**) The result of applying adaptive thresholding. (**c**) The result of applying a Gabor filter and binary thresholding. (**d**) The final particle segmentation result.

The PAA is a method for segmenting an ROI at the pixel level to identify undissolved solutes. The number of pixels identified as undissolved particles was used as a feature called the ‘Number of particles’. The numbers of fine particles and clumps of particles were counted separately and added together. First, fine particles were segmented in the ROI by applying adaptive thresholding [27]. Adaptive thresholding is an algorithm that adaptively determines a threshold value based on the local conditions. We used a method that calculates the local threshold value as a weighted average of neighboring pixels using a Gaussian kernel, with the block size set to 151 and the c value set to 10. The final threshold value was determined by subtracting the c value from the threshold value, which was calculated as the weighted average of the neighboring pixels. Because the segmentation performance is poor for large clumped particles, the clumps of particles are segmented in the ROI by applying a Gabor filter [28] and binary thresholding. The sum of the number of fine particles and pixels identified as clumps of particles is used as the final feature. The PAA sequence is shown in Figure 7.

#### 3.4.4. Superposition Analysis

**Figure 8:**
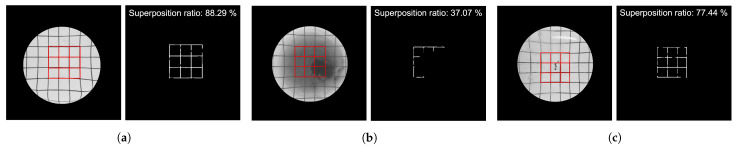
For each figure, the left panels show the predicted checked background image using the detected check pattern and the right panels show the superposition result of the predicted checked background image and the detected check pattern. (**a**) DS, (**b**) US1, (**c**) US2 case examples.

SA is a method for obtaining the superposition of the check pattern in an ROI image and the check pattern in a checked background image. First, a check pattern is detected in the ROI image. The following approaches are used to detect the check pattern in the ROI image. The image is binarized using the adaptive thresholding method and Canny edge detection [29] is employed to locate the edges. Subsequently, the progressive probabilistic Hough transform [30,31] is utilized to detect the lines, and morphological expansion and closure operations [32] are applied to ultimately detect the check pattern. The detected check pattern is used to predict the original checked background image. Instead of predicting the entire checked background image, only the central region corresponding to the ROI was predicted as a grid shape with nine parts. The nine grids were predicted as follows: the horizontal pixel intensity values of the detected check pattern image were examined to select six y-coordinates in descending order. Similarly, the vertical pixel intensity values of the detected check pattern image were examined to select six x-coordinates in descending order. When selecting the representative coordinate values, only one coordinate per check pattern line was used; therefore, a non-maximum suppression process was performed. This was achieved by disregarding values within ±25 pixels of the selected coordinate pixels. Finally, representative x- and y-coordinate values were obtained for each of the six lines. Using only the four coordinate values closest to the center, a grid-shaped check pattern was predicted in nine parts. This is considered the check pattern in the checked background image, and the superposition with the detected check pattern is obtained as a feature called the ‘Superposition ratio’. The SA results are presented in Figure 8.

### 3.5. Classifier Design

We trained a classifier using nine handcrafted features extracted from the data and classified the solutions based on their solubility. We named the network, which performs a series of processes to extract features using GHA, RPA, PAA, and SA, trains a DNN classifier, and classifies solutions, ‘ASSNet’. ASSNet utilizes a DNN model as the classifier, which is trained using the cross-entropy [33] loss function and the Adam optimizer [34] with a learning rate of 0.001.

The DNN model architecture consisted of four fully connected layers with ReLU activation [35], and the final layer utilized the softmax function. The architecture consisted of the following layers: an input layer with (9, 64) neurons, a hidden layer with (64, 128) neurons, a second hidden layer with (128, 256) neurons, and a final output layer with (256, 3) neurons. Each layer was implemented using the PyTorch nn.Linear module. The size of each layer was selected based on prior domain knowledge and empirical experimentation. The feature values obtained using the proposed analysis method were normalized to a mean of 0 and variance of 1.

## 4. Experimental Result

### 4.1. Dataset

**Figure 9:**
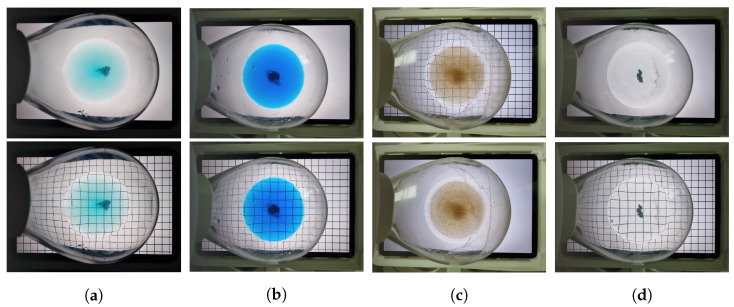
Examples of training datasets with varying amounts of non-dissolving solutes. (**a**) CuSO${}_{4}$, (**b**) CuOAc, (**c**) CuBr, (**d**) Pd(OAc)${}_{2}$.

We utilized a training dataset [4] consisting of copper sulfide (CuSO${}_{4}$), copper acetic acid (CuOAc), copper bromide (CuBr), and palladium acetic acid (Pd(OAc)${}_{2}$) as the solutes and deionized (DI) water as the solvent. The solvent–solute combinations of the training dataset used in the experiment are listed in Table 2; there were 151 sample combinations in total. As the dataset obtained from the direct experimentation was not abundant, augmentation was applied to the training dataset using vertical flips, horizontal flips, and vertical and horizontal flips. In addition, solution images were captured from the two background images. The final number of images in the training dataset was 1208, which was obtained by multiplying the number of sample combinations by eight. Examples of the training dataset are shown in Figure 9.

**Figure 10:**
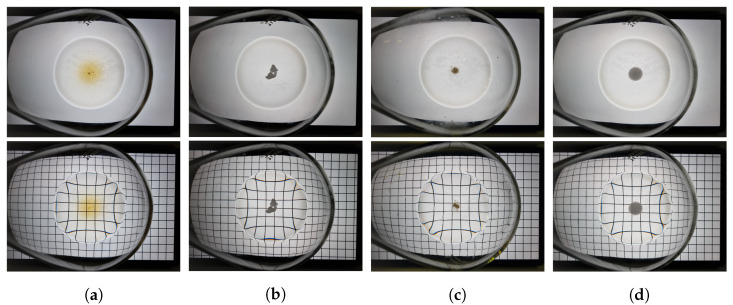
Examples of the test datasets; (**a**) 2-bromo-4-phenylpyridine, (**b**) 4-methoxyphenol, (**c**) naphthalic anhydride, (**d**) 4,4′-bis(α,α-dimethylbenzyl)diphenylamine.

For the test dataset, we used 2-bromo-4-phenylpyridine, 4-methoxyphenol, naphthalic anhydride, and 4,4′-bis(α,α-dimethylbenzyl)diphenylamine as solutes and toluene, methylene chloride, and hexane as solvents, which are actively used at industrial sites. The solvent–solute combinations used in the test dataset are listed in Table 3, and there are 97 sample combinations in total. Augmentation was not applied to the test dataset when validating our model. However, when conducting the k-fold cross-validation [36] by combining the training and test datasets, we applied the same augmentation techniques used for the training dataset to the test dataset. Examples of the test dataset are shown in Figure 10.

**Figure 11:**
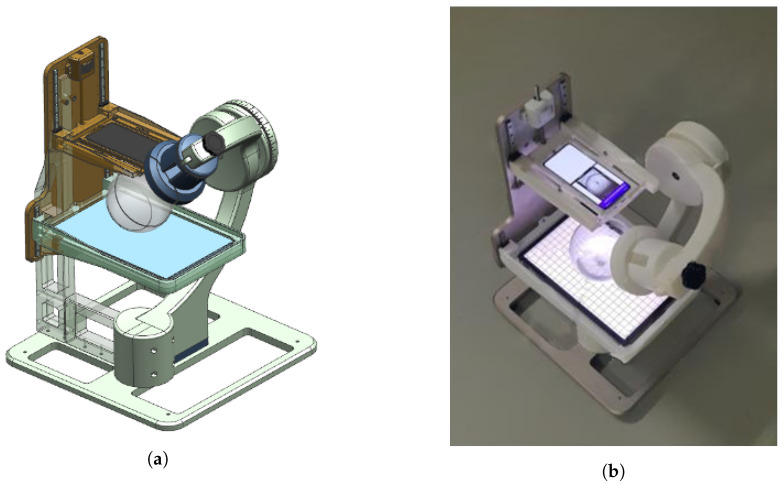
Equipment used for data acquisition. (**a**) Equipment modeling, (**b**) actual equipment.

The datasets are labeled DS, US1, and US2, based on their solubility. Each case is illustrated in Figure 1. The dataset images were captured in the experimental environment shown in Figure 11, where the flask containing the solution was placed on a tablet displaying a background image, and captured using the rear camera of a mobile phone. The experimental environment was the same as that used by Kim et al. [4].

### 4.2. Comparison with End-to-End Learning Model

To demonstrate the validity of the handcrafted features in our task, we compared ASSNet, which uses handcrafted features, with an end-to-end learning approach. Our solution dataset was preprocessed with zero padding to create a square image and center crop for training on different end-to-end learning models. Solution images captured on a checked background were not used in training. All end-to-end models were fine-tuned using pre-trained weights, and cross-entropy was used as the loss function, with optimization performed using the Adam optimizer and a learning rate of 0.001. All models were trained for 100 epochs, and all k-fold validations were conducted with 10 folds.

As shown in Table 4, the end-to-end learning models were overfitted to the training dataset and performed poorly on the test dataset and k-fold cross-validation. For certain tasks, such as analyzing the solubility of a solution and working with small-sample datasets used in the industry, manually extracting features and classifying them has performed well.

### 4.3. Classification Results Based on Classifier

When training a classifier with handcrafted features to classify solutions, it shows high classification performance, even when using linear SVM [10,42] as an additional classifier alongside a DNN. The experiment was conducted using a linear SVM model with a regularization parameter, ‘C’, set to 30. Although SVM had a shorter training time, it showed lower performance than the DNN in terms of the training dataset, test dataset, and k-fold cross-validation experiments. The experimental results based on this classifier are listed in Table 5.

### 4.4. Ablation Studies

**Figure 12:**
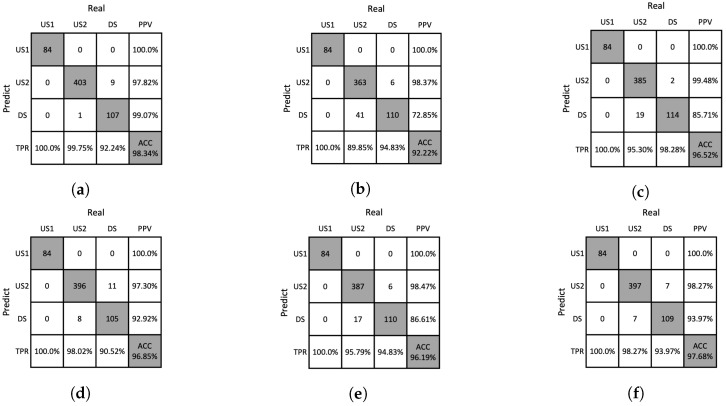
Confusion matrices resulting from ablation studies of DNN on the training dataset. (**a**) w/o Moiré removal, (**b**) w/o GHA, (**c**) w/o RPA, (**d**) w/o PAA, (**e**) w/o SA, (**f**) our method.

**Figure 13:**
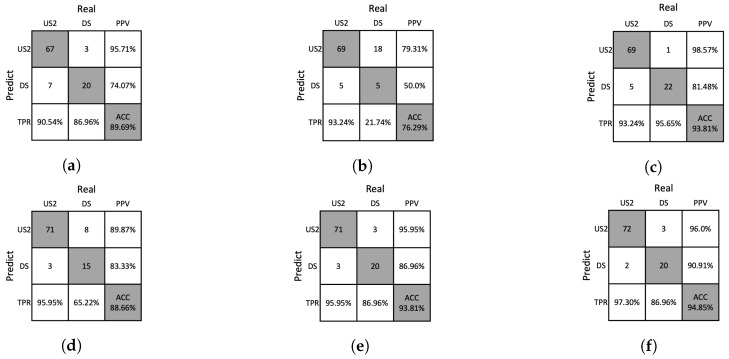
Confusion matrices resulting from ablation studies of DNNs on the test dataset. (**a**) w/o Moiré removal, (**b**) w/o GHA, (**c**) w/o RPA, (**d**) w/o PAA, (**e**) w/o SA, (**f**) our method.

**Figure 14:**
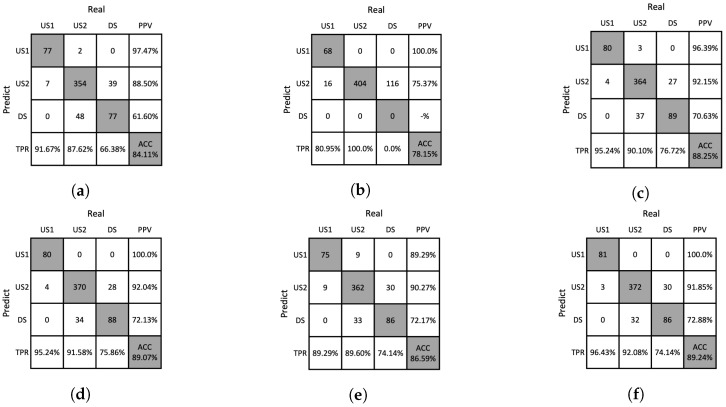
Confusion matrices resulting from ablation studies of SVM on the training dataset. (**a**) w/o Moiré removal, (**b**) w/o GHA, (**c**) w/o RPA, (**d**) w/o PAA, (**e**) w/o SA, (**f**) our method.

**Figure 15:**
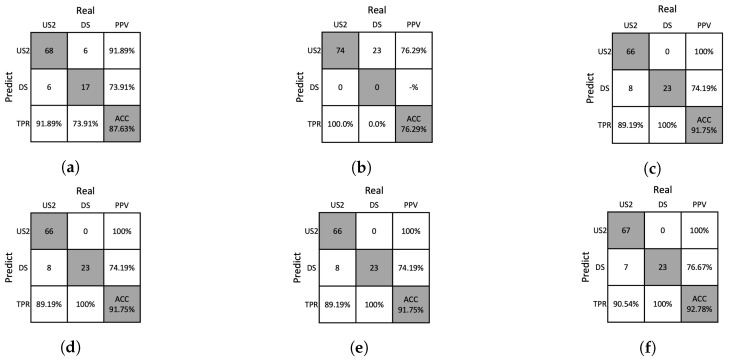
Confusion matrices resulting from the ablation study of SVM on the test dataset. (**a**) w/o Moiré removal, (**b**) w/o GHA, (**c**) w/o RPA, (**d**) w/o PAA, (**e**) w/o SA, (**f**) our method.

We conducted an ablation study to analyze the influence of handcrafted features obtained from the removal of Moiré patterns and the proposed analysis methods. The ablation studies for the DNN and SVM classifiers are presented in Table 6 and Table 7, respectively. It was found that the performance tended to decrease when the Moiré patterns were not removed or when the proposed analysis methods were not used. The confusion matrices for the ablation study are shown in Figure 12, Figure 13, Figure 14 and Figure 15, which allows us to investigate the extent to which the Moiré removal and the proposed analysis methods affect the classification of solutions. The results between US1 and US2 reveal the impact on the turbidity measurement, whereas the results between US2 and DS provide insights into particle detection. For instance, when the GHA was not applied, the classification performances of the US2 and DS solutions were significantly lower, indicating the importance of the GHA in particle detection.

The evaluation metrics used in the experiments are the true positive rate (TPR) and positive predictive value (PPV). The TPR is the proportion of actual positive cases correctly identified as positive by the model, and it is calculated as TPR = TP/(TP + FN). The PPV is the proportion of positive predictions made by the model that are actually true positives, and it is calculated as PPV = TP/(TP + FP). True positives (TP), false positives (FP), and false negatives (FN) are defined as follows:

- TP: Number of cases in which the model correctly predicts a positive outcome for a positive case.
- FN: Number of cases in which the model predicts a negative outcome for a positive case.
- FP: Number of cases in which the model predicts a positive outcome from a negative case.

## 5. Conclusions

In this study, we propose a method for automatically determining the solubility of a solution using only a tablet or mobile phone. Previous studies have attempted to automatically measure the solubility using the brightness of the solution or an end-to-end learning approach. However, these methods have limited ability to discriminate the presence of undissolved fine particles and are not suitable for environments with insufficient datasets. Therefore, we propose ASSNet, a network for classifying solutions by extracting handcrafted features from solution images captured on a white or checked background and trained using a DNN classifier. The proposed method can also determine the presence or absence of fine particles owing to its precisely designed feature extraction pipeline.

We compared the end-to-end learning method with ASSNet, and found that the performance of ASSNet was superior, as shown in Table 4. Additionally, the experimental results in Table 6 and Table 7 demonstrate the validity of the extracted features and the classification performance according to the feature extraction method. To validate the performance of the proposed method, we utilized datasets labeled DS, US1, and US2, which contain many images that are difficult to classify, even for humans.

We expect that our method, if applied to a system that automatically changes the solution in a flask, can achieve an end-to-end automated process. The proposed method has the potential to improve the accuracy of solubility determination and automate related processes, thereby facilitating research in various fields.

## Figures and Tables

**Table 1:** Handcrafted features.

Feature Name	Background Type	Analysis Method
MMG	White	GHA
MSG	White	GHA
SMG	White	GHA
SSG	White	GHA
Minimum value	White	RPA
Curvature	White	RPA
MSE	White	RPA
Number of particles	White	PAA
Superposition ratio	Checked	SA

**Table 2:** Combination of the solutes and solvents in the training dataset.

Solute	Solvent	No. Samples
CuSO${}_{4}$	DI water	43
CuOAc	DI water	41
CuBr	DI water	30
Pd(OAc)${}_{2}$	DI water	37

**Table 3:** Combination of solutes and solvents in the test dataset.

Solute	Solvent	No. Samples
2-bromo-4-phenylpyridine	Toluene	10
4-Methoxyphenol	Toluene	65
Naphthalic anhydride	Toluene	5
Naphthalic anhydride	Methylene chloride	3
Naphthalic anhydride	Hexane	2
4,4′-bis(α,α-dimethylbenzyl)diphenylamine	Toluene	12

**Table 4:** Comparison of various models.

Model	Training Dataset Acc	Test Dataset Acc	k-Fold Avg Acc
ResNet18 [37]	98.11 ± 0.43	54.29±3.05	86.71±0.47
ResNet34 [37]	98.87±0.43	55.01±3.40	84.28±1.42
InceptionV3 [38]	96.36±0.31	61.43±3.43	78.10±1.38
DenseNet121 [39]	98.84±0.26	82.02±1.42	85.92±0.80
MobileNetV2 [40]	98.28±0.30	71.78±2.06	83.98±1.05
MobileNetV3(small) [41]	98.54±0.25	79.46±1.67	83.01±0.68
MobileNetV3(large) [41]	99.27±0.32	80.89±1.74	84.82±0.50
ASSNet (Ours)	97.29±0.38	93.20±1.88	94.00±1.25

**Table 5:** Comparison between DNN and SVM.

Model	Training Dataset Acc	Test Dataset Acc	k-Fold Avg Acc
DNN	97.29±0.38	93.20±1.88	94.00±1.25
SVM	89.24±0.00	92.78±0.00	90.69±0.11

**Table 6:** Ablation studies on DNN.

Moiré Removal	GHA	RPA	PAA	SA	Training Dataset Acc	Test Dataset Acc	k-Fold Avg Acc
-	✓	✓	✓	✓	98.34	89.69	89.29
✓	-	✓	✓	✓	92.22	76.29	87.78
✓	✓	-	✓	✓	96.52	93.81	91.52
✓	✓	✓	-	✓	96.85	88.66	89.80
✓	✓	✓	✓	-	96.19	93.81	91.61
✓	✓	✓	✓	✓	97.68	94.85	92.12

**Table 7:** Ablation studies on SVM.

Moiré Removal	GHA	RPA	PAA	SA	Training Dataset Acc	Test Dataset Acc	k-Fold Avg Acc
-	✓	✓	✓	✓	84.11	87.63	86.29
✓	-	✓	✓	✓	78.15	76.29	81.96
✓	✓	-	✓	✓	88.25	91.75	90.32
✓	✓	✓	-	✓	89.07	91.75	90.32
✓	✓	✓	✓	-	86.59	91.75	89.72
✓	✓	✓	✓	✓	89.24	92.78	90.53

## Data Availability

The data are not publicly available due to privacy restrictions.
